# Does Cardiac Contractility Modulation Therapy Reduce Atrial Fibrillation Burden?

**DOI:** 10.19102/icrm.2022.13104

**Published:** 2022-10-15

**Authors:** Jae Wook Shin, Rami Atoot, Marissa Heyer, Sameer Jamal

**Affiliations:** ^1^Department of Medicine, Division of Cardiology, Rutgers New Jersey Medical Schoolm, Newark, NJ, USA; ^2^Hackensack Meridian School of Medicine, Hackensack, NJ, USA; ^3^Division of Cardiac Electrophysiology, Department of Cardiology, Hackensack Meridian School of Medicine, Hackensack, NJ, USA

**Keywords:** Atrial fibrillation, cardiac contractility modulation

## Abstract

Cardiac contractility modulation (CCM) is an implantable technology approved by the U.S. Food and Drug Administration and intended for heart failure patients without a cardiac resynchronization therapy indication. CCM leads to reduced heart failure hospitalizations and improvements in exercise tolerance and quality of life. There are a lack of data examining the impact of CCM therapy on atrial fibrillation (AF) burden. We report the case of a 65-year-old man with a history of paroxysmal AF, hypertension, hyperlipidemia, and carotid artery stenosis who presented with newly diagnosed ischemic cardiomyopathy with a left ventricular ejection fraction (LVEF) of 20%–25%. He underwent coronary artery bypass graft surgery for triple vessel disease with an improvement in LVEF to 40% after 4 months of guideline-directed medical therapy. Due to clinical heart failure and paroxysms of AF, he underwent CCM device and implantable loop recorder (ILR) implantation. His LVEF improved to 60%, and the ILR showed no AF. We postulate multiple mechanisms to explain the negligible burden of AF.

## Background

Cardiac contractility modulation (CCM) is a therapy that delivers electrical stimuli during the cardiac absolute refractory period, increasing the cytosolic calcium level, which results in an increase in left ventricular (LV) force production.^1^ The Optimizer^®^ Smart device (Impulse Dynamics, Marlton, NJ, USA) is an implantable, commercially available technology approved by the U.S. Food and Drug Administration that delivers CCM therapy intended for patients with clinical heart failure in the absence of a cardiac resynchronization therapy (CRT) indication.^2^ CCM therapy was evaluated against optimal medical therapy in the Evaluate Safety and Efficacy of the Optimizer^®^ System in Subjects with Moderate-to-Severe Heart Failure (FIX-HF-5C) study and led to both reduced heart failure hospitalizations and improvements in exercise tolerance and quality of life. While the initial CCM device required atrioventricular synchrony, the current iteration can deliver therapy in those with rate-controlled atrial fibrillation (AF).^3^ There is a paucity of data examining the impact of CCM therapy on AF burden.

## Case presentation

A 65-year-old man with a history of hypertension, hyperlipidemia, and carotid artery stenosis presented to the emergency department with 3 weeks of orthopnea, paroxysmal nocturnal dyspnea, and progressive shortness of breath. An echocardiogram showed newly diagnosed severe LV dysfunction with an ejection fraction (LVEF) of 20%–25%, moderate mitral regurgitation, and mild left atrial dilation. Left heart catheterization demonstrated triple vessel disease, and the patient ultimately underwent coronary artery bypass graft (CABG) surgery.

Postoperatively, he was continued on guideline-directed medical therapy (GDMT). He developed paroxysms of symptomatic AF, lasting several hours at a time and developing days after his CABG surgery. While he did not have a previous diagnosis of AF, he recalled comparable symptoms for months before his CABG surgery and had not sought medical attention. As his CHA_2_DS_2_-VASc score was 4 points, he was started on apixaban (Eliquis^®^; Bristol-Myers Squibb, New York, NY, USA) for thromboembolic risk reduction and amiodarone for sinus rhythm maintenance.

His LVEF remained severely depressed at 20% 12 days after his CABG surgery, and he was discharged to a rehabilitation center. Over the next 4 months, he was continued on GDMT and his echocardiogram showed an increase in LVEF from 20% to 40%, and he no longer required implantable cardioverter-defibrillator therapy. Despite this, he continued to have symptoms of congestive heart failure and was not a candidate for cardiac resynchronization therapy. Therefore, a decision was made to proceed with Optimizer^®^ Smart CCM device implantation to improve his functional status. In addition, he continued to report rare episodes of palpitations, albeit less frequently than before his CABG procedure and amiodarone initiation. A formalized assessment of rhythm burden was not performed during this initial postoperative period. Rather, a decision was made to implant a LINQ implantable loop recorder (ILR) (Medtronic, Minneapolis, MN, USA) for monitoring his arrhythmia and determining the AF burden. Amiodarone was discontinued at the time of CCM device and ILR implantation.

After CCM therapy, his functional status improved, and he started walking 0.5–1.5 mi daily after 2 weeks of CCM therapy. His EF improved to 60% 3 months after implantation of the Optimizer^®^ Smart CCM device. His ILR has been remotely and continuously monitored for approximately 1.5 years. The ILR has not shown any AF, and he has not required any additional rhythm-controlling therapy. The patient also described an increased sense of well-being and a greatly improved quality of life.

## Decision-making

**Figure 1:**
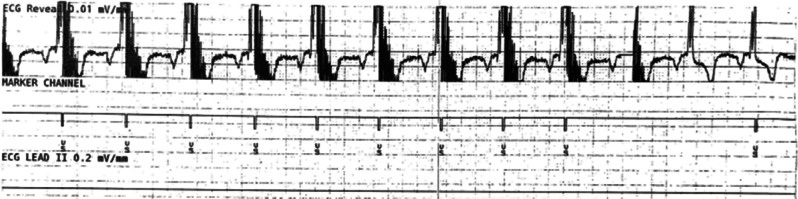
Implantable loop recorder showing delivered cardiac contractility modulation therapy during the absolute refractory period.

**Figure 2:**
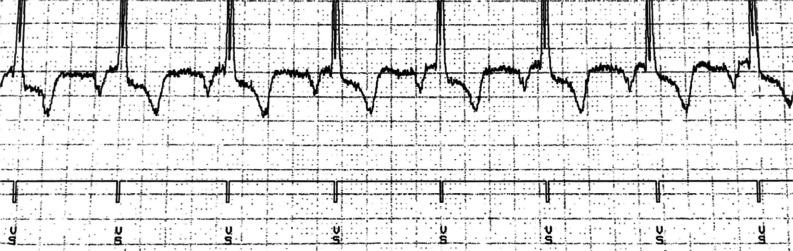
Implantable loop recorder showing native electrical activity without cardiac contractility modulation therapy.

Although the patient’s LVEF improved to 40% on optimal GDMT, he continued to demonstrate clinical heart failure. While he did not exhibit clinical recurrence of AF, it was determined that continued arrhythmia surveillance was necessary. After discussion with the patient, ILR and CCM devices were implanted. Sharp signals just after the QRS complexes in all but the last few complexes of **Figure 1** demonstrate the delivery of CCM during the absolute refractory ventricular period. In contrast, the end of the tracing reveals more normal-appearing QRS complexes without CCM therapy, and **Figure 2** shows more of the patient’s native heart rhythm without the delivery of CCM.

## Conclusion

This case report demonstrates that CCM therapy can improve clinical heart failure in patients who are ineligible for CRT and remains a novel therapy with increasing rates of use and adoption. The AF burden in our patient is negligible since the initiation of CCM therapy. We also demonstrate the delivery of CCM therapy on ILR. To the best of our knowledge, there is no study evaluating the relationship between AF burden and CCM device implantation. In our example, a few mechanisms may explain the findings. CCM therapy may reduce AF burden as an impact of LVEF improvement. CCM may directly mitigate or reverse remodeling of atrial myocytes, thereby preventing the structural and electrical changes that perpetuate AF. CCM therapy could cause reverse modeling of left atrial myocytes, which decreases left atrial pressure and prevents left atrial remodeling that can precipitate AF. These findings are hypothesis-generating, and additional investigation into the association between AF and CCM therapy is needed.
